# Coherent beam combining of two all-PM thulium-doped fiber chirped pulse amplifiers

**DOI:** 10.1007/s12200-024-00117-3

**Published:** 2024-05-28

**Authors:** Bo Ren, Hongxiang Chang, Can Li, Tao Wang, Kaikai Jin, Jiayi Zhang, Kun Guo, Rongtao Su, Jinyong Leng, Pu Zhou

**Affiliations:** 1 College of Advanced Interdisciplinary Studies, National University of Defense Technology, Changsha, 410073 China; 2 Nanhu Laser Laboratory, National University of Defense Technology, Changsha, 410073 China; 3 Hunan Provincial Key Laboratory of High Energy Laser Technology, National University of Defense Technology, Changsha, 410073 China

**Keywords:** Coherent beam combining, Thulium-doped fiber laser, High-average power, Chirped pulse amplifier

## Abstract

In this paper, we report a coherent beam combining (CBC) system that involves two thulium-doped all-polarization maintaining (PM) fiber chirped pulse amplifiers. Through phase-locking the two channels via a fiber stretcher by using the stochastic parallel gradient descent (SPGD) algorithm, a maximum average power of 265 W is obtained, with a CBC efficiency of 81% and a residual phase error of *λ*/17. After de-chirping by a pair of diffraction gratings, the duration of the combined laser pulse is compressed to 690 fs. Taking into account the compression efficiency of 90% and the main peak energy proportion of 91%, the corresponding peak power is calculated to be 4 MW. The laser noise characteristics before and after CBC are examined, and the results indicate that the CBC would degrade the low frequency relative intensity noise (RIN), of which the integration is 1.74% in [100 Hz, 2 MHz] at the maximum combined output power. In addition, the effects of the nonlinear spectrum broadening during chirped pulse amplification on the CBC efficiency are also investigated, showing that a higher extent of pulse stretching is effective in alleviating the spectrum broadening and realizing a higher output power with decent combining efficiency.

**Graphical Abstract:**

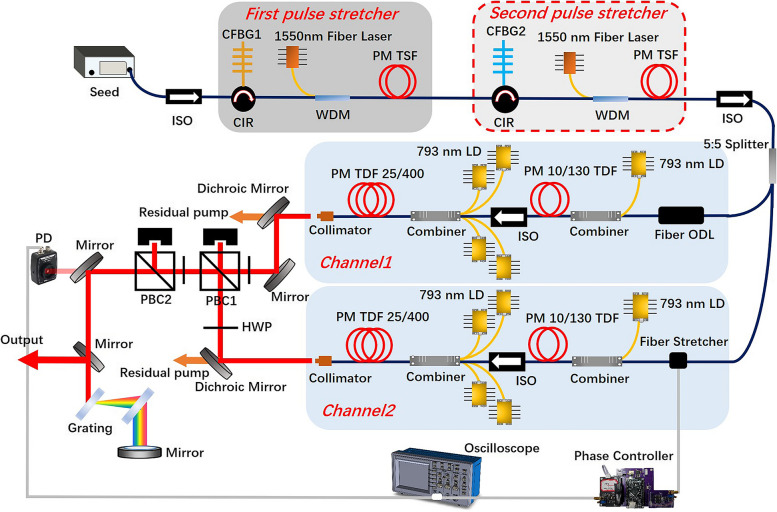

## Introduction

Over the last decade, the high-power ultrafast laser with an operation wavelength extending to the 2.0 μm range has been attracting increasing research interests, mostly driven by its applications in various fields such as remote sensing, material processing, medical care, and mid-infrared broadband supercontinuum generation [1–6]. Specifically, the thulium-doped fiber (TDF) chirped pulse amplifier (CPA) has been recognized as a promising candidate for generating high average power ultrafast lasers at 2.0 μm, thanks to the merits of fiber lasers in terms of high conversion efficiency, efficient heat dissipation and excellent beam quality [7–11]. With the recent emergence of large mode area (LMA) thulium-doped photonic crystal fiber, 2 μm ultrafast laser with up to 1-kW average power has been realized from TDF-CPA system [12]. Whereas owing to the tailored structure of the LMA fiber, previous demonstrations concerning high power ultrafast TDF amplifiers were mostly implemented with free-space coupled configuration, leading to a compromise of the compactness and the robustness of the laser system [13–16]. Moreover, due to the spectral phase distortions and thermal lens effect induced by the remarkable water vapor absorption lines at ~ 1900 nm, the amplified laser is prone to be deteriorated by strong beam deformation and nonlinear phase accumulation [17]. As such, an all polarization maintaining (PM) fiberized TDF amplifier is highly preferred for realizing a high quality and reliable operation of the laser source.

It should be noted that in an all-fiber laser amplifier, the active and passive fibers with relatively small diameter for fusion splicing and long transmission length for connection would cause significant nonlinear effects, hindering the scale of the output power [18, 19]. At present, the maximum output average power from the all-PM fiber CPA in the 2.0 μm range is 314 W [20], which is much lower than that from the free-space coupled counterparts. To further enhance the laser power, an alternative scheme is the coherent beam combining (CBC), which partitions the laser signal into multiple parallel amplification channels and subsequently coherently combines them into a single output beam with a power scaling factor being nearly the number of channels employed, thereby effectively mitigating the impact of nonlinearities and thermal effects of a single fiber amplifier [21–23]. A precondition for high efficiency CBC is the phase-locking between channels, which is usually realized by real-time controlling of phase modulators via active phase control algorithms such as multi-frequency dithering and stochastic parallel gradient descent (SPGD) algorithm [24–27]. Currently, significant progress has been achieved in the 1.0 μm range [28–32], and the highest average power of 10 kW [28] and single pulse energy of 32 mJ [31] were respectively obtained from coherently combined multiple fiber CPA channels. Regarding the 2.0 μm range, there are also efforts concerning the CBC [33–35], the correspondingly realized maximum pulse energy is up to 1.65 mJ [36] in femtosecond fiber lasers. Nevertheless, the employment of free-space coupled amplification channels in those demonstrations renders the system fragile and cumbersome, as well as difficult to extend the combining channels. More compact CBC of ultrafast lasers at 2.0 μm based on the all-fiber amplification channels has not yet been reported.

In this work, we experimentally demonstrated a two-channel CBC system based on all-PM TDF-CPA. The phase locking was realized through incorporating into one of the channels a high-speed fiber stretcher that was controlled by the SPGD algorithm, while an optical delay line inserted into the other channel was manually adjusted to guarantee the coherent interference between combined beams. A maximum combined average power of 265 W was obtained with the de-chirped pulse duration of 690 fs.

## Experimental setup

**Fig. 1:**
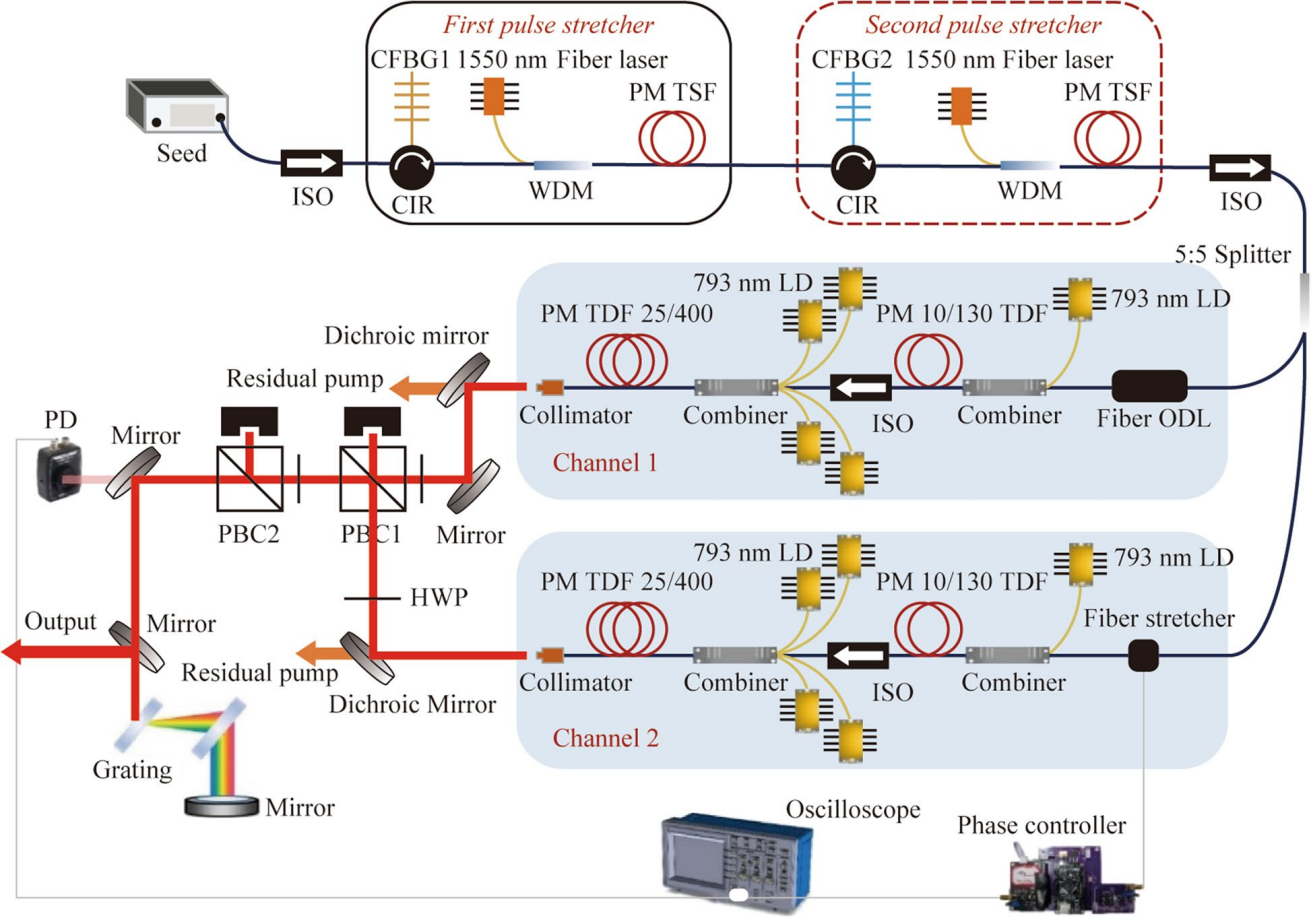
Experimental setup of the CBC system based on two channels of all-PM TDF-CPA. ISO: isolator, CFBG: chirped fiber Bragg grating, CIR: circulator, WDM: wavelength division multiplex, LD: laser diode, ODL: optical delay line, PBC: polarization beam combination, HWP: half wave plate, PD: photoelectric detector

**Fig. 2:**
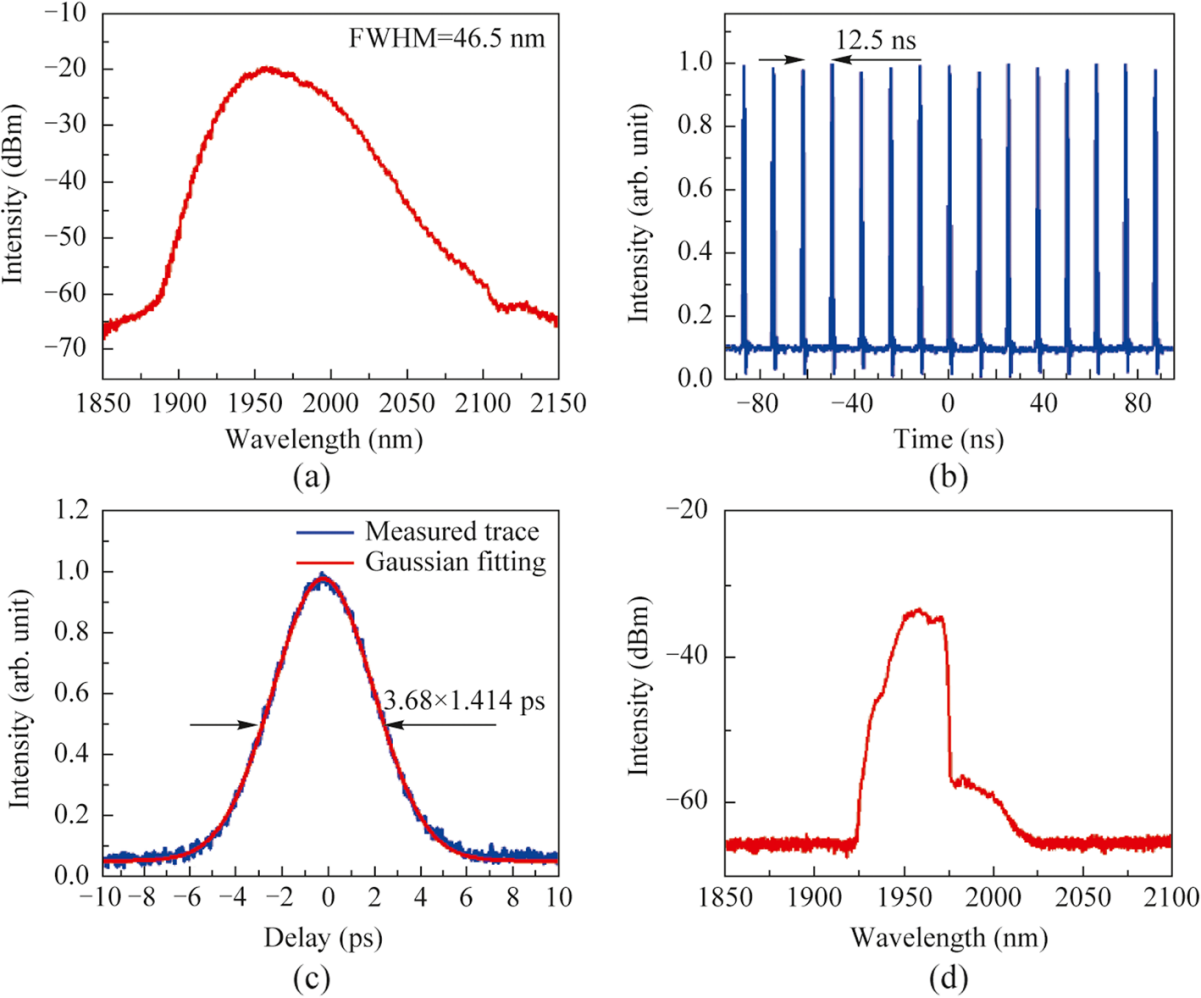
**a** Optical spectrum, **b** pulse train and **c** pulse duration of the seed laser; **d** spectrum of the laser signal after the pre-amplification with core-pumping

The experimental setup of the coherently combined system based on two channels of all-PM TDF-CPA is illustrated in Fig. 1. The seed laser was a commercial mode-locked TDF oscillator with a central wavelength of 1957 nm and a 3-dB bandwidth of 46.5 nm (Fig. 2a). In addition, it emitted 180 mW positively chirped pulses with a repetition frequency of 80 MHz and a duration of 3.68 ps, as respectively shown in Fig. 2b and c. The pulse duration was temporally stretched to around 800 ps by using two stages of stretchers to manage the nonlinear effects. Each pulse stretcher consists of a circulator and a PM chirped fiber Bragg grating (CFBG) with a reflectivity of 15% and a positive second-order dispersion of + 34.159 ps^2^. Meanwhile, in order to compensate for the insertion loss (~ 10 dB) caused by the CFBG, a pre-amplifier constructed with a 4-m-long PM thulium-doped single-mode fiber (TSF) with a core/cladding diameter of 9/125 μm was employed to booster the signal power to ~ 300 mW, with the core-pumping by a high-power 1550 nm fiber laser though a wavelength division multiplexer (WDM). Figure 2d shows the measured laser spectrum after the pre-amplification, and it is observed that the 3-dB bandwidth was narrowed to 25.5 nm, as a result of the limited reflection bandwidth of the CFBG. After passing through an isolator and a 5:5 splitter, the laser signal was divided and respectively launched into two separate amplification channels that have similar configuration. Both channels include two stages of amplification with the same length of the active fiber and pigtails to ensure commensurate accumulation of the nonlinear phase shift. In the first amplification stage, a 2.5-m-long double-clad PM-TDF with a core/cladding diameter of 10/130 μm, a core numerical aperture (NA) of 0.15, and an absorption coefficient of 4.7 dB/m at 793 nm was utilized to enhance the signal power to 6 W, which was realized by the pumping of a 793 nm multi-mode laser diode via a combiner. Between the amplification stages, an isolator was employed to block the back-scattered light. The main amplifier was constructed with four commercial 793 nm laser diodes that deliver a total maximum power of 400 W, a (6 + 1) × 1 pump/signal combiner, and a 5-m-long commercial PM-LMA-TDF which has a core/cladding diameter of 25/400 μm, a core NA of 0.09, and an absorption coefficient of 2.4 dB/m at 793 nm. Moreover, the PM-LMA-TDF was coiled in a runway type and temperature controlled by an aluminum heat sink, which was coordinately water-cooled with a temperature of ∼12°C. Subsequently, the amplified laser of the two channels were respectively emitted from a fiber collimator that incorporated with a cladding power stripper, and then passed through a dichroic mirror to separate from the residual pump.

To realize CBC of the two amplification channels, a fiber stretcher with a total length of 10 m and a stretch accuracy of 0.2 μm/V was incorporated into channel2 for real time phase locking between the channels. In addition, an electronically controlled fiber optical delay line (ODL) was inserted into channel1 to compensate for the optical path difference with a compensation range of 3 cm and an accuracy of 100 μm, enabling the realization of high fringe contrast for the coherent interference of the combined beams. The laser beams were orthogonally coincided at a polarization beam combiner (PBC1), prior to which half wave plates (HWPs) were utilized to adjust the polarization directions and guarantee orthogonal polarization overlapping. The combined beam was then passed through another HWP and PBC2 for diagnosing the combining efficiency. In principle, coherent polarization combining of two orthogonally polarized beams would generate a single linearly polarized beam with high polarization extinction ratio (PER). After that, a reflective mirror was used to reflect the combined beam to a folded Treacy grating compressor, which was constructed by utilizing a transmission diffraction grating pair with line density of 800 l/mm for pulse de-chirping. Moreover, the transmitted laser signal from the mirror was detected by a photodetector, which cooperated with an oscilloscope and a phase controller for real-time controlling the fiber stretcher based on the SPGD algorithm.

## Results and discussion

**Fig. 3:**
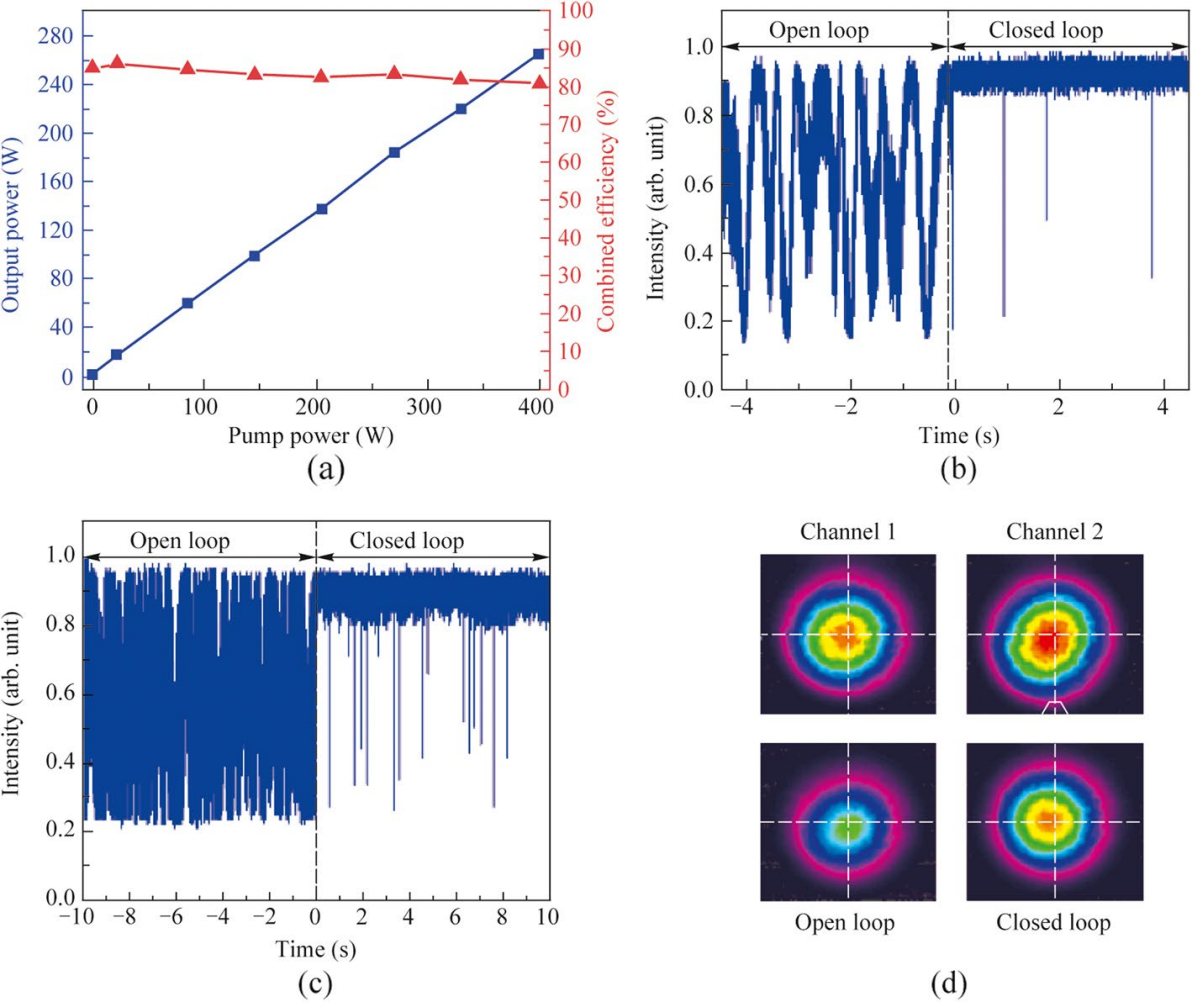
Output characteristics of the CBC system: **a** output power and combining efficiency versus the pump power of the main amplifiers; normalized temporal intensity fluctuations of the combined laser before and after phase locking (i.e., open loop and closed loop) at the output power of **b** 1.6 W and **c** 265 W, respectively; **d** beam profiles of respectively the output of the two amplification channels, and that of the CBC with the open and closed loop under the operation power of 265 W

The efficiency and output power of the CBC were recorded and depicted in Fig. 3a. When the main amplifiers were unpumped, the transmitted power through PBC2 was 1.6 W with phase locking. By calculating the ratio of the transmitted output power to the input power through PBC2, the corresponding combining efficiency was ~ 86%. Compared with previous demonstrations, the relative lower combining efficiency was mainly attributed to the limited compensation accuracy of the ODL. With increasing the pump power of the main amplifier, the combining efficiency was tardily decreased to 81%, and a pump power limited output power of 265 W was obtained. Figure 3b and c show respectively the normalized temporal intensity fluctuations of the combined laser with open and closed loop at the combined output power of 1.6 and 265 W. It is noted that at each of the measured power level, the ODL in channel1 was mannually adjusted to maintain a highest possible contrast of the intensity fluctuation under the open loop regime, as the thermal effect in the main amplifier would induce drifting of the optical delay and weaken the interference effect. It can be seen from the figure that in the open loop regime, the contrast of the intensity fluctuations with zero and maximum pumping of the main amplifiers are roughly commensurate, indicating that the coherence of the laser changed hardly after the power scaling. Whereas in the condition of closed loop as shown in Fig. 3c, the intensity fluctuations minimized significantly and its characteristic parameters can be leveraged to estimate the residual phase error of the CBC system [37], the residual phase error at the maximum output power of 265 W was calculated to be ~ *λ*/17. Moreover, occasionally dropping of the combined intensity (more than 50%) is observed from Fig. 3c, and it is primarily attributed to the restricted voltage range of the phase controller and the slow response of the fiber stretcher. Regarding the output beam profiles, the randomly variated profile of the combined beam with open loop immediately changed into a stable Gaussian shape upon closing the controlling loop at the maximum combined power of 265 W, as shown in Fig. 3d.

**Fig. 4:**
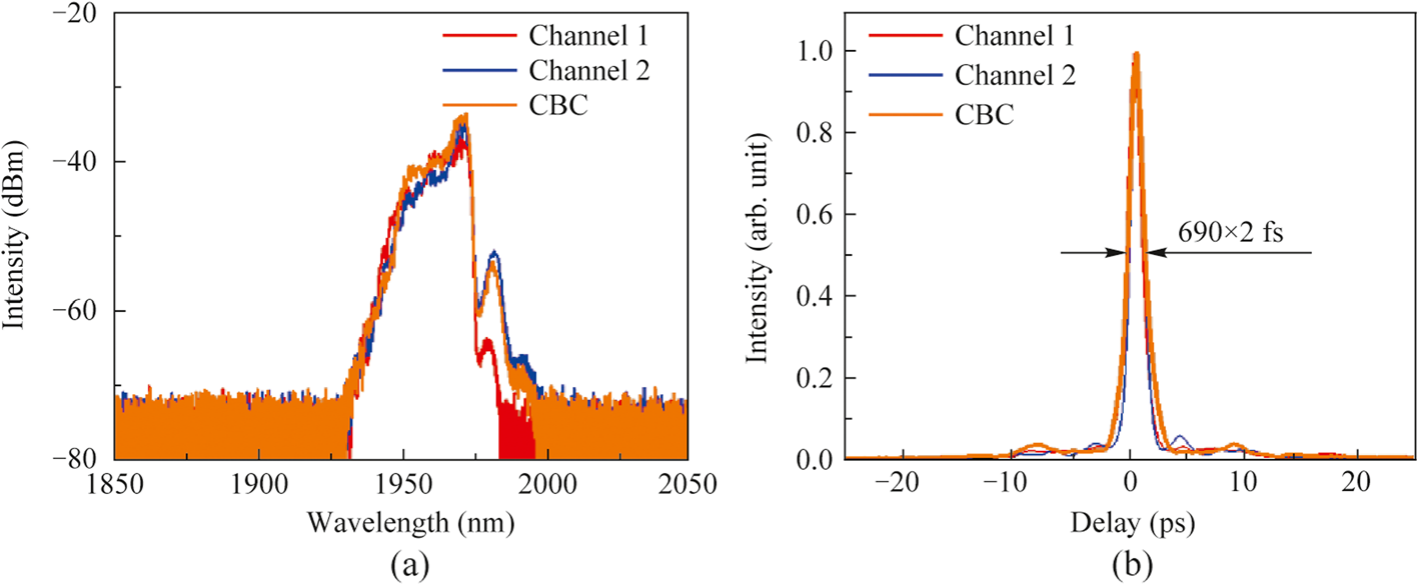
Output characteristics of the single amplification channels and the CBC at the output power of 265 W: **a** spectra; **b** pulse autocorrelation traces after compression

Figure 4a shows the optical spectra of the single amplification channels and the CBC at the maximum output power of 265 W. Thanks to the larger extent of pulse stretching, the spectral broadening induced by the nonlinear effect was trivial and the laser spectra roughly preserved that of the seed before splitting. The B-integral of the two amplification channels were respectively calculated as 0.94 and 1.09, indicating that the laser pulse was basically amplified in a quasi-linear manner and the slight difference in profile of the output spectra of the two channels, while the coherently combined spectrum basically mimicked the broader version. The corresponding pulse autocorrelation traces after compression are depicted in Fig. 4b. It is noted that in order to realize a shorter compressed pulse, the high-order dispersion of CFBG2 in Fig. 1 was coordinately adjusted via an engineered temperature profile to compensate for the accumulated nonlinear phase. With a superior compression efficiency of 90%, the Lorentz fitted pulse width was 690 fs with a main peak energy proportion of 91%, corresponding to a single pulse energy of 3 μJ and a peak power of 4 MW.

**Fig. 5:**
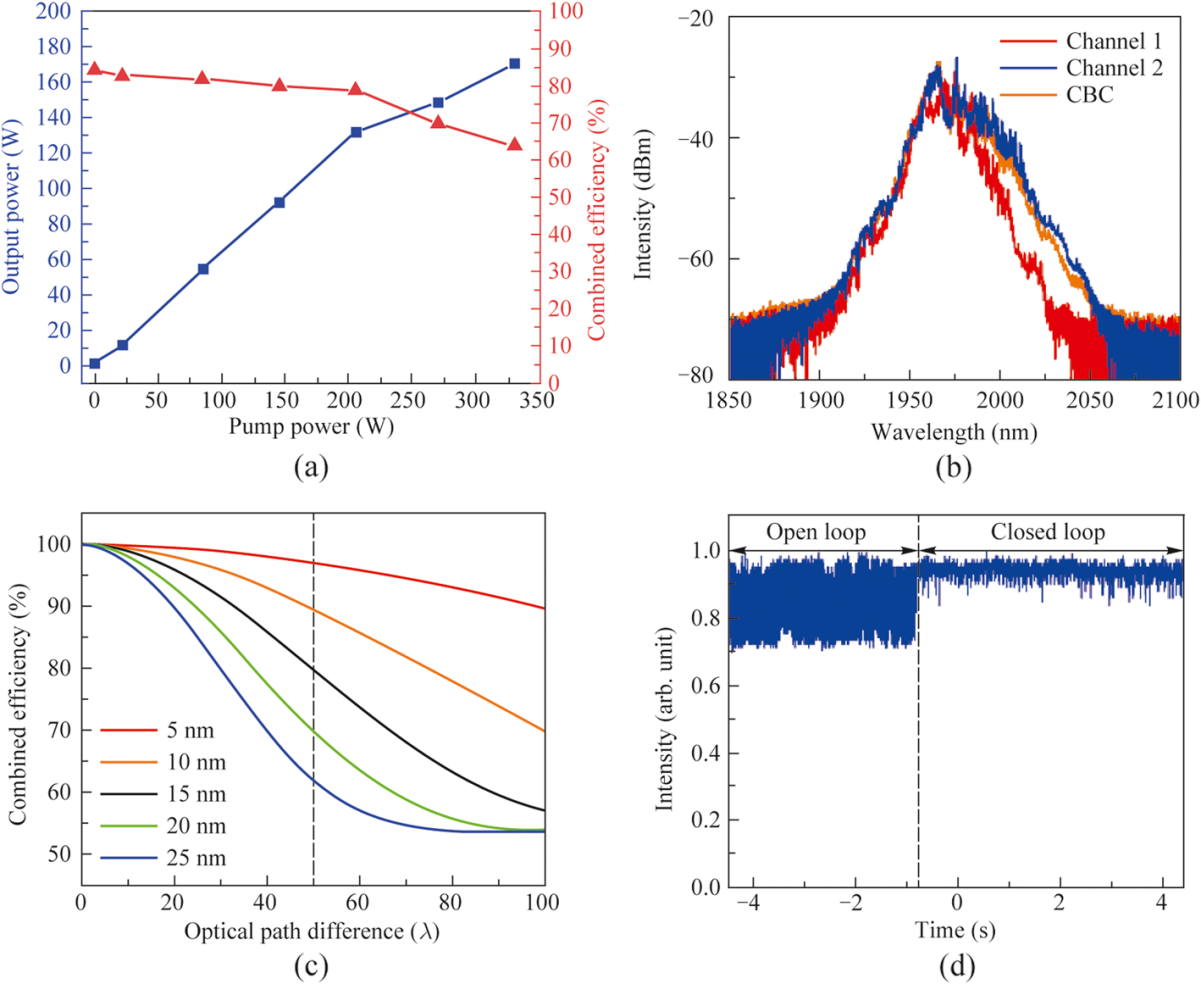
Output characteristics of the CBC system with single pulse stretcher: **a** output power and combining efficiency versus the pump power of the main amplifiers; **b** output spectra of the two amplification channels and the CBC with closed loop at the output power of 172 W; **c** the calculated combining efficiency versus the optical path difference under different 3 dB bandwidth of the spectrum; **d** normalized temporal intensity fluctuations of the combined laser before and after phase locking (i.e., open loop and closed loop) at the output power of 172 W

Unlike conventional CBC of narrow linewidth laser with long coherence length, the ultrafast laser generally involves nonlinear amplification and significant spectral broadening, rendering it more challenging to realize a high combining efficiency. In subsequent experiments, the impact of spectrum broadening during chirped pulse amplification and the controlling accuracy of the optical path difference on the CBC efficiency was further investigated. By removing the second pulse stretcher in Fig. 1, the pulse duration of the seed laser was stretched to only ~ 400 ps. As shown in Fig. 5a, with the increase of the pump power, the combining efficiency significantly diminished to 65% at a combined output power of 172 W. As shown in Fig. 5b, the spectra of the amplification channels and the CBC with closed loop were broadened significantly compared with that of the seed laser. In addition, the amplified output power of the two channels were respectively 124 and 143 W, corresponding to a calculated nonlinear phase shift accumulation of 1.64 and 1.84 rad, indicating a considerably enhanced nonlinear effects during the amplification process.

Additionally, according to the analysis in Ref. [38], the spectral broadening could decrease the coherence length and thus the combining efficiency under a certain optical path difference, and can be calculated as [38]:

1 $$\eta = \frac{1}{2} + \frac{1}{2}\frac{{\sqrt {P_{1}^{2} + P_{2}^{2} + 2P_{1} P_{2} \left\{ {2\exp \left[ { - \frac{1}{8\ln 2}\left( {\frac{\Delta \omega }{{c_{0} }}\Delta l} \right)^{2} } \right]\cos^{2} \left( {\frac{{\omega_{0} }}{{c_{0} }}\Delta l} \right) - 1} \right\}} }}{{P_{1} + P_{2} }},_{{}}$$

where *η* is the combining efficiency, *P*_1_ and *P*_2_ are respectively the peak power of the two channels, Δ*ω* is the full width at half maximum of the pulse spectrum, *ω*_0_ is the central angular frequency of the spectrum, *c*_0_ is the speed of light in vacuum, and Δ*l* is the optical path difference. It should be noted that Eq. (1) is based on the assumption that other factors that affect the combining efficiency have been completely eliminated or compensated. Figure 5c demonstrates the calculated combining efficiency versus the optical path difference under different 3-dB bandwidth of the spectrum. It is observed that the impact of optical path difference drifting between the two channels on the combining efficiency would become increasingly significant with the broadening of the laser spectrum. Considering an ODL accuracy of 100 μm (approximately 50*λ*), the system combining efficiency would diminish from 97% to nearly 60% with the 3-dB bandwidth of the spectrum broadened from 5 to 25 nm, agreeing well with the experimental results. Figure 5d shows the normalized temporal intensity fluctuations of the combined laser before and after phase-locking (i.e., open loop and closed loop) at the output power of 172 W. It is observed that the spectral broadening induced weakening of the interference effect between the main amplifiers is remarkable as shown in the open loop trace, and the corresponding residual phase error with phase-locking was calculated to be *λ*/13. Therefore, except for improving the controlling accuracy of the optical path, another intuitive scheme to increase the combining efficiency is to manage the nonlinear effects in the fiber amplifier to mitigate the spectral broadening or distortion of the amplified laser.

**Fig. 6:**
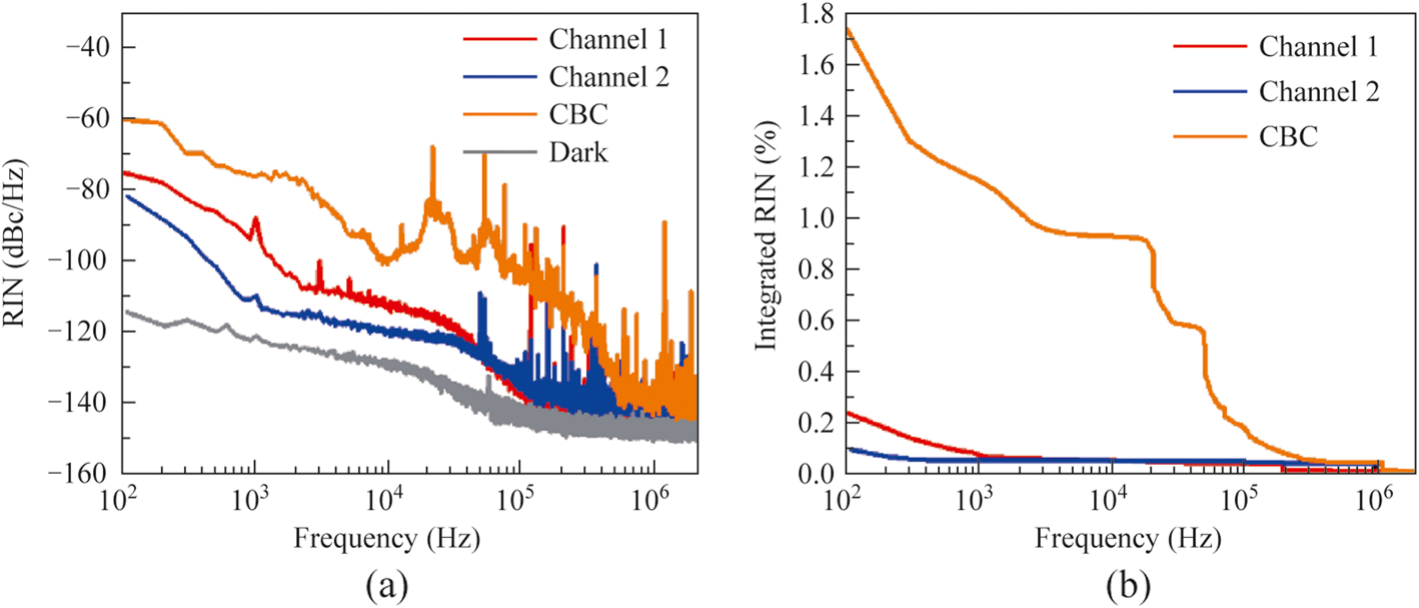
Noise characteristics of the single amplification channels and the CBC at the output power of 265 W: **a** relative intensity noise spectra; **b** integrated relative intensity noise

To further characterize the noise properties of the CBC system, the relative intensity noise (RIN) at the maximum combined power of 265 W was examined from 100 Hz to 2 MHz by using a signal analyzer, a 12.5 GHz photodetector, and a 2.5 MHz low-pass filter was used to avoid the detector from being saturated by the signal at the repetition frequencies. The measured RIN spectra are shown in Fig. 6a, in which the RIN of the single amplification channels are about the same level at frequencies higher than 50 kHz and reaches to a noise plateau of − 140 dBc/Hz at the frequency of 2 MHz. Whereas at frequencies < 50 kHz, the RIN of channel 2 is lower than that of channel 1, mostly owing to the variations of pump power and thermal effects between the channels. Moreover, the RIN spectrum of the combined laser exhibited an overall increase of ~ 20 dB at frequencies < 300 kHz, primarily attributed to the remainder phase noise of the CBC system and the electronic noise of the controlling loop [28]. Additionally, the integrated RIN of channel 1, channel 2, and the CBC at the maximum output power were respectively 0.24%, 0.09%, and 1.74%, as shown in Fig. 6b.

## Conclusion

In conclusion, we have experimentally demonstrated a CBC system based on two thulium-doped all-PM fiber chirped pulse amplifiers. By actively controlling the relative phase with a fiber stretcher based on the SPGD algorithm, the coherent combining efficiency was ~ 81% with an estimated residual phase error of *λ*/17 at the maximum output power of 265 W. After being compressed by a pair of diffraction gratings, the duration of the combined laser pulse was measured to be 690 fs. Taking into account the compression efficiency of 90% and a main peak energy proportion of 91%, the corresponding peak power was calculated to be 4 MW. An examination of the noise characteristics manifested that the CBC would degrade the laser RIN, which was estimated to be 1.74% with an integration frequency range from 100 Hz to 2 MHz at the maximum output power. Additionally, the impact of nonlinear effects induced spectrum broadening during chirped pulse amplification on the loss of CBC efficiency was further investigated, and the results indicated that a higher extent of pulse stretching is effective in alleviating the nonlinear spectrum broadening and maintaining a preferable combining efficiency at higher output power. It is anticipated that higher power and energy femtosecond laser output can be realized through increasing the number of amplification channels and optimizing the controlling loop.

## Data Availability

The data that support the findings of this study are available from the corresponding authors upon reasonable request.
